# Birth Preparedness among Women Who Gave Birth in the Last Twelve Months in Jardega Jarte District, Western Ethiopia

**DOI:** 10.1155/2019/6473725

**Published:** 2019-04-01

**Authors:** Tiruneh Asrat, Negga Baraki, Nega Assefa, Getachew Alemkere

**Affiliations:** ^1^Easte Wollega Zonal Health Office, Nekemte, Oromia National Regional State, Ethiopia; ^2^College of Health and Medical Sciences, Haramaya University, Harar, Ethiopia; ^3^Department of Pharmacology and Clinical Pharmacy, College of Health Science, Addis Ababa University, Addis Ababa, Ethiopia

## Abstract

**Background:**

Lack of preparedness for rapid action in the event of obstetric complications was the major problem contributing for delay in receiving skilled obstetric care. This study aimed to assess birth preparedness and factors associated with it among women who gave birth in the last 12 months preceding the survey in Jardega Jarte district, Western Ethiopia.

**Methods:**

A community-based cross-sectional study was conducted from January to February 2016. A total of 581 women who gave birth recently were randomly selected for an interview. Data were entered and analyzed using SPSS version 21. Binary logistic regression was performed to identify predictive factors. Statistical significance was declared at p<0.05.

**Results:**

From 581 questionnaires distributed, 570 were completed making the response rate 98%. The mean age was 28 with a standard deviation of 5 years. Ninety percent of the respondents were rural in residency. The average family size was 6 with a range of 13. Majority of the respondents were grand multipara, 261(45.6%). Despite the majority (69.3%) of the respondents reported as they made arrangement for birth, only 27.5% of them were well-prepared for birth and its complication management. Urban residency (AOR=3.4, 95% CI: 1.7-6.9), primipara (AOR=5.12, 95% CI: 2.4-10.8), history of obstetric complication (AOR=4.05, 95% CI: 2.4-7.75), and attending antenatal care (AOR=2.9, 95% CI: 1.67-5.16) were independently associated with preparation for birth and its complication.

**Conclusion:**

This study revealed that only about a quarter of pregnant women were well-prepared for delivery and complication management. Urban residencies, history of past obstetric complications, availing antenatal care, primipara, and absence of an under-five child in the household during recent delivery were predictors of birth preparedness. On the other hand, availing health service to such rural areas, giving more attention to the grand multiparous mothers with large family size will be important interventions to prevent pregnancy-related complications. Such efforts would benefit from accessing antenatal care and family planning services.

## 1. Introduction

Decreasing maternal death was topmost global health agenda since the previous two decades. World Health Organization (WHO) estimated that each year above half million mothers die from avoidable pregnancy-related complications. The situation is most awful for Sub-Saharan Africa region. In the region one in every 16 women dies from pregnancy-related causes as compared to one in 2,800 in developed regions during the mother's lifetime [1].

It is estimated that greater than 90% of maternal deaths could be avoided even in the low-income countries condition [2, 3]. However, the encumbrance is still high. Every year three million early neonatal deaths, four million stillbirths, and nearly half million maternal deaths are still related to complications of pregnancy worldwide. This figure is much higher than the five million projected deaths for HIV/AIDS, tuberculosis and malaria combined. The majority (99%) of expected worldwide maternal death occurs in resource-limited settings, the Sub-Saharan Africa accounting for more than half of the reported death rate [4].

Birth preparedness is considered as a useful and practical intervention with several advantages. In particular, it can increase use of skilled services to avoid the potential unexpected events during pregnancy and help them to plane for the required backup [5]. The level of maternal morbidity and mortality in Ethiopia was among the highest and accounted 21% of all deaths of women aged 15–49 years. Majority of this death could be averted if a woman and her family had a birth and emergency preparedness plan [6].

Detrimental delays in seeking care were mainly attributed to lack of awareness to the danger signs of complication during and after pregnancy or delivery. Studies made clear that most of pregnant women and their caregivers do not know the danger signs of pregnancy-related complications [7, 8]. The WHO estimates that 300 million women in the developing world suffer from short-term to long-term illness brought about by pregnancy and childbirth. This is because of several reasons one of which is inadequacy or lack of birth and emergency preparedness [9].

Birth preparedness encompasses the process of planning for normal birth and anticipating the actions needed in case of an emergency. It can be measured by the mothers' knowledge on identifying danger signs and their preparation to take measures during emergency and normal obstetric care. Among the preparations expected form the mothers were (i) identification of a trained (skilled) birth attendant, (ii) identification of health facility, (iii) identification of mode of transport, (iv) saving money, and (v) Identified blood donor in case needed for obstetric emergency [1–9].

According to growing body of evidence in developing countries, birth preparedness can be influenced by the sociodemographic characteristics of women, the cultural context, and socioeconomic factors. According to a study from Burkina Faso women who saved money for the coming pregnancy were 2.48 times more likely to get a skilled birth assistant than their nonplanned counterparts [10]. A study in southeastern Nigeria showed that variables like age, parity, and educational and marital status are the best predictor of awareness towards the concept of birth preparedness. Place of residence was not a good predictor of awareness of birth preparedness according to this study [11]. A study in Adegirat of Ethiopia identified maternal education and marital status as a strong predictor of preparation [12]. A similar study in Aleta Wondo district, southern Ethiopia, revealed that educational level and residence as a predictor of birth preparedness [13].

Despite the fact that birth preparedness is essential for improvement of maternal and child health, studies conducted in some parts of Ethiopia revealed a low birth preparedness level [12, 13]. In addition, a little or none is known about the current magnitude and influencing factors in the Western part of the country. This study is therefore focused on assessing factors associated with birth preparedness among women who gave birth in the last twelve months in Jardega Jarte West District. The results of the study will provide valuable information for the design of programs and interventions to improve maternal and neonatal health in the district.

## 2. Methodology

### 2.1. Study Area and Period

The study was conducted in Jardega Jarte district from January 1 to February 2, 2016. Jardega Jarte district is one among nine districts found in Horo Guduru Wollega zone of Oromia region, Western Ethiopia. Alibo is the district's town, which is at about 380 KM from Addis Ababa, the capital city. Administratively the district is divided into 18 rural and 3 urban kebeles. As projected from Central Statistical Agency (CSA) 2007 report, the district has a total of 56,432 in 2014. Oromo ethnicity is the dominant native of the district, with the Christian dominant religion followers. The life of the population mainly depends on seasonal farming [5]. Currently, there are three public health centers and 18 health posts in the district. All health centers provide 24-hour services.

### 2.2. Study Design

A community-based cross-sectional study design was used on women who gave birth within the last 12 months preceding the survey (from December 31, 2015, to January 1, 2016). Those women who were a permanent resident of the study area (Living more than six months in the study area) were included while women, who are critically ill or could not talk or listen were excluded from the study.

### 2.3. Sample Size and Sampling Methods

A single population proportion formula was used to calculate the sample size. Proportion (P) was taken to be 22% from another study in the country [12]. The margin of error is 5%, 95% confidence level, with a design effect of 2 and 10% nonresponse rate; the final the sample size becomes 581. Since the matrix in the urban and rural area is extremely heterogeneous, all the kebeles in the district were stratified into urban and rural strata's. Then one urban and 9 rural kebeles were randomly selected for the study by lottery method, with the assumption that the probability for a woman to be included in the study is the same within the strata's wherever she delivered. After that, the census was conducted from January 1 to 10, 2016, in those selected kebeles to register all women who were within 12 months of postdelivery. Community health workers working in those selected kebeles conducted the census under health extension workers supervision. Accordingly, 855 women were registered. The calculated sample size was proportionally allocated to the kebeles and a simple random sampling technique (lottery method) was used to select the specific samples.

### 2.4. Data Collection Instrument

A pretested structured interviewer-administered questionnaire was taken from the safe motherhood questionnaire developed by maternal and neonatal health program of JHPIEGO and adapted according to the local context and the objectives of the study [14].

#### 2.4.1. Data Collectors and Supervisors

The data collectors were females who knows the study area and the local language well and who had a certificate of grade ten or above. Accordingly, twelve females were selected. Five nurses supervised the data collection (one supervisor for two kebeles). Both the interviewers and supervisors were given three days training before the actual work about the aim of the study, study procedures, data collection techniques, art of interviewing, and the issue of confidentiality. The practical exercise was made through peer interview.

Community health workers who conducted the census assisted the data collectors in finding the direction of the household traced by the census. During the actual data collection, each supervisor supervised two data collectors, in two kebeles. Every day, the supervisors checked all the filled questionnaires for completion, clarity, and consistency. Then, the principal investigator had also checked randomly selected responses. Incomplete and unclear questionnaires were returned to the interviewers to get it completed for the next day.

### 2.5. Data Quality Control

Recall bias was minimized by involving women who gave birth recently (in the last 12 months). Standard questionnaire of safe motherhood was taken and modified based on study interest. The instrument was pretested on 30 (5%) of the samples in a district (Amuru) not included in the study. Modifications were made after pretest. The questionnaire was translated into the local language, Afan Oromo and back retranslated to English to check for consistency. The interviewers and supervisors were given three days training before the actual work. Close supervision during data collection, with daily feedback and proper cleaning before and after entry, was made seriously.

### 2.6. Study Variables

#### 2.6.1. Dependent Variable

*Birth Preparedness*. Birth preparedness is the process of planning for normal birth and anticipating the actions needed in case of an emergency [15]. Birth preparedness for a pregnant mother in the current study context was to mean (I) identifying a trained (skilled) birth attendant, (II) identifying health facility for emergency and normal obstetric care, (III) identifying mode of transport for delivery and/or for an obstetric emergency, (IV) saving money, and (V) identifying blood donor in case needed.

#### 2.6.2. Independent Variables

*Sociodemographic and Economic Variables*. Maternal age, marital status, ethnicity, religion, fixed income source, family size, residence, educational status (husband/women), and occupation (husband/women).

*Obstetric Characteristics*. Gravidity, parity, antenatal care use, and past obstetric history (any obstetric danger sign and complication in a lifetime, history of stillbirth).

### 2.7. Data Processing and Analysis

The collected data was coded, checked, cleaned, and analyzed using SPSS version 21. Descriptive analysis was used to summarize the finding. Then those mothers who mentioned at least three of the five birth preparedness lists were considered “well-prepared”. These were the identification of health facility for emergency and normal delivery, arranging transportation, and saving money. The remaining pregnant women were considered “less prepared”. A binary logistic regression model was used to test association. Crude and adjusted odds ratio (OR) and the respective 95% CI (confidence interval) was used to determine the magnitude of the association. Variables that have p<0.25 in the univariate model were included in the final multivariable logistic regression model. Finally, the association was declared at P<0.05.

### 2.8. Operational Definitions

*Recently gave birth*: a woman who gave birth within the last twelve months (from January 1/2015 to December 31/2016) preceding the study.

*Health extension workers*: female health workers, who are working at health post (the smallest level of health institution in Ethiopia) after getting one-year health science training [16].

*Birth preparedness*: delineates the roles (plans/actions) of policymakers, facility managers, care providers, communities, families, and women in ensuring that women and newborns receive timely appropriate and effective care [15].

*Skilled birth attendant*: persons with midwifery skills who can manage normal deliveries, diagnosis, and manage or refer obstetric complications (physicians, health officers, nurses, and midwives) [17].

*Permanent residence:* according to Ethiopian CSA (Central Statistical Agency), a person who lives at least six months in the study area [5].

*Kebele*: smallest administration unit in Ethiopia [5].

*Well-prepared for birth and its complication*: according to this study if a woman identified health facility for emergency and normal delivery, arranged transportation, and saved money during pregnancy for the recent delivery.

*Obstetric danger signs*: signs that may not be actual obstetric complications, but symptoms that are easily identified by nonclinical personnel.

*Key obstetric danger signs* are those that are common, can easily be recognized, and are signs of serious obstetric complications that require immediate medical intervention.

## 3. Results

### 3.1. Sociodemographic Characteristics of the Study Participants

From 581 questionnaires distributed, 570 were completed making the response rate 98%. The mean age was 28 with a standard deviation of 5 years. Ninety percent of the respondents were rural in residence. Oromo by ethnicity and Orthodox by religion were dominant among the study participants with a proportion of 86.5% and 34.9%, respectively. Similarly, the majority (95.3%) of them were in the marital union during the study. About 320(56%) of the study participants attended primary education. Twenty-five percent of the participants have any form of fixed income obtained in cash or in kind (Table 1).

### 3.2. Experiences of Respondents on the Recent Pregnancy and Delivery

About 235 (41.2%) of the study participants were below six-month postdelivery while 335 (58.8%) gave birth before six months preceding the survey. Above half (53.9%) of the participants had under-five children in the house during pregnancy of the recent delivery. Only 164 (28%) of the respondents attend antenatal care visit at least once during their recent pregnancy. Majority of the participant, 470 (82.5%), gave birth at home in their recent delivery. One hundred and sixty-five (28.9%) of the study participants have a history of obstetric complication in the past. Major complications experienced were a severe headache 88 (21.5%) followed by vaginal bleeding 74 (18%) and blurred vision 86 (21%), respectively (Table 2).

### 3.3. Knowledge of Obstetric Danger Sign among Study Participants

Despite the majority of the mothers reported as they know, below half of them exactly identified the danger signs (report versus exact identification of all key danger signs: 69.5% versus 31.48%, 60.4% versus 19.5% for pregnancy, and labor and birth, respectively). On the other hand however above half of those who reported as they know the danger signs for postpartum complication (54.9%) above half of them were able to identify all the key danger signs (57.2%) (Table 3).

### 3.4. Level of Birth Preparedness

Majority, 395 (69.3%), of the respondents reported that they have made some arrangement for the birth of their recent baby. However, only 157 (27.5%) of the study participants were well-prepared for birth and its complications (Table 4).

### 3.5. Factors Associated with Birth Preparedness

Primipara compared to grand multipara women were more likely to prepare themselves for birth and its complications (AOR=5.13, 95% CI: 2.42-10.83). Women living in urban were three times more likely to prepare themselves than their counterparts (AOR=3.44, 95% CI: 1.69-6.96). Women having previous obstetric complication were four times prepared than their counterparts (AOR=4.3, 95% CI: 2.3-7.75). In addition, women who had attended antenatal care during pregnancy of the recent delivery were two times more likely to prepare themselves than those who did not attend (AOR=2.94, 95% CI (1.67-5.16). A woman who had an under-five child in the house during pregnancy of recent delivery was less likely to prepare themselves than those who did not have (AOR=0.206, 95% CI: 0.122-0.35). Having a fixed income source (COR=4.37, 95% CI: 2.9-6.5), husband occupation (COR=3.57, 95% CI: 2.3-5.37), and husband age were associated with birth preparedness in the univariate analysis, but failed to associate in the multivariate analysis (Table 5).

## 4. Discussion

Birth preparedness is a central element of antenatal care aimed at reducing risky delays in pursuing emergency obstetric care so as to aver unwanted maternal and fetal outcomes [1]. In this study, several important findings were observed. Slightly higher than a quarter of pregnant women were well-prepared for delivery and emergency obstetric care. Urban residencies, history of past obstetric complications, availing antenatal care, being pregnant for the first time, and presence of an under-five child in the household during recent delivery were predictors of birth preparedness. The finding of this study is majorly consistent with previous studies [12, 18] and reinforce efforts to increase birth preparedness should focus on accessing and delivering health education through antenatal care and family planning services.

Our study revealed that despite the majority (69.3%) of the respondents reported as they made arrangement for birth, only 27.5% of them were well-prepared for birth and its complication management. This result is slightly higher than the study conducted in Sidama (17%) [13] and Wolyita (18.3%) zones [18] of Ethiopia. On the other hand, it is lower than an Indian report, 47.8% [12], and Ugandan report, 35% [19]. Difference in the study settings as well as sociocultural differences may contribute for the observed difference.

In our study, being urban resident had a significant association with birth preparedness. This could be because of that the urban residents had better access to health information and maternal health services as compared with rural counterparts. This finding is not in line with a study conducted in Southern Nigeria where no difference in residence was observed [11].

Average family size of the study participants was 6 with a range of 13. As a result, the majority of the respondents were grand multipara, 261(45.6%). Women who were primipara compared to grand multipara were more likely to be prepared for birth and its complication. This is in agreement with a study conducted in Uganda [20] and Ethiopia [18]. This could be because of freshness that might force to give ears secondary to the high-risk perception of such women than those who had experience. This shows that increasing risk perception might help in improving BPACR. In addition, an increase in family size may increase routines for family care and thus decrease the risk perception tendency and the quality of birth preparedness and complication management readiness.

In this study, about 29% of the study participants had a history of obstetric complication in the past. Women with a history of past obstetric complication were more likely to be well-prepared than their counterparts (AOR=4.3, 95% CI: 2.4-7.75). This finding is similar to a study done in Adigrat and Wolayta, Ethiopia [18, 20]. This could be due to the reason that those women anticipate serious complications based on their previous experiences of risk perception.

Above half (53.9%) of the participants had under-five children in the house during pregnancy of the recent delivery. Presence of under-five child in the household during pregnancy of the recent delivery has an independent negative association with birth preparedness (AOR=0.206, 95% CI: 0.122-0.35); this may be due to the fact that women take care of this under-five child rather than preparing themselves for the coming delivery. This may signify the need for family planning education in line with the mothers' health.

Twenty-eight percent of the respondents attend antenatal care at least once during their recent pregnancy. In the current study, similar to studies conducted in India [19], Adegirat [12] and southern Ethiopia [13, 18], women attending ANC were well-prepared compared to their counter parts. This signifies the importance of the ANC setups in improving pregnancy-related awareness. Since we do not have health related Medias to reach Ethiopian mothers so far, the role of the ANC remains crucial. It is probably the only means for the rural women to access accredited health information.

It can be assumed that the financial issues might have an effect on the birth preparedness. Accordingly studies had estimated that greater than 90% of maternal deaths could be avoided even in the low-income countries condition [2, 3]. In this study mothers having a fixed income source (COR=4.37, 95% CI: 2.9-6.5) were more likely to get prepared as per the univariate analysis. However, this association disappeared in the multivariate model. In addition, husband occupation (COR=3.57, 95% CI: 2.3-5.37) and husband age were associated with birth preparedness in the univariate analysis. The first can be explained in terms of thee education status of the husbands as almost all of the farmers in our country context were uneducated. Although it is difficult to justify, the latter intern means that the husband's aged 40 or less were more likely to help their wife's to prepare for birth. Unfortunately, both of the variables were failed to associate in the multivariate analysis (Table 5).

Potential weakness related to this study is temporal ambiguity. Women who delivered prior to an interview may have difficulty in recalling preparations they made or services they used during that pregnancy and childbirth. In this study, women below 12-month postpartum period were interviewed, thus reducing recall bias

The advantage of asking women who are currently pregnant about birth preparedness is that these actions will be immediate and therefore easier to report accurately. However, since they have not completed their pregnancies yet, they may not make an arrangement for birth. In addition, there may be a variation in their service use experience. Therefore, they are only able to provide information based on their plan to use those services.

## 5. Conclusions

Although more than half of women reported as they know the occurrence of danger sign attributed to pregnancy, about a quarter of them were able to identify the key danger signs. Women living in urban areas, primipara, history of past obstetric complications, and antenatal care follow-up history were independent positive predictors for birth preparedness. However, the presence of an under-five child in the household during recent delivery was identified as an independent negative predictor for birth preparedness. This study revealed that only about a quarter of pregnant women were well-prepared for delivery and obstetric complication management. Urban residencies, history of past obstetric complications, availing antenatal care, primipara, and absence of an under-five child in the household during recent delivery were predictors of birth preparedness. On the other hand, availing health service to such rural areas, giving more attention to the grand multiparous mothers with large family size will be important interventions to prevent pregnancy-related complications. Such efforts would benefit from accessing antenatal care and family planning services. In general, according to the current and several other studies, expansion and capacitation of the antenatal care clinic service will have a paramount worth in averting pregnancy-related complications.

## Figures and Tables

**Table 1:** Sociodemographic characteristics of study participants' who gave birth recently, in Jardega Jarte District, Oromia Region, western Ethiopia, from January 1 to February 2, 2016.

Variables	Number (n=570)	Percent
*Age in years*, Mean (± standard deviation)	28 ± 5	
15-24	150	26.3
25-34	318	55.8
≥3	102	17.9
*Educational status*		
Illiterate	158	27.7
Primary	320	56.1
Secondary and above	92	16.1
*Religion*		
Protestant	168	29.5
Orthodox	199	34.9
Muslim	111	19.5
Catholic	92	16.1
*Ethnicity*		
Oromo	493	86.5
Amhara	38	6.7
Tigray	22	3.9
Gurage	17	3.0
*Marital status*		
Currently in marital union	543	95.3
Currently not in marital union	27	4.7
*Have fixed income source*		
Yes	144	25.3
No	426	99.7
Average family size (Range)	6 (0-13)	

**Table 2:** Experiences of respondents who gave birth recently in Jardega Jarte district, Oromia Region, western Ethiopia, from January 1 to February 2, 2016.

Variables	Frequency (N=570) (%)
*Parity*	
Primipara,	64 (11.2%)
Multipara	245 (43%)
Grand Multipara.	261(45.6%)
*Under-five children during the recent delivery*	
Present	307(53.9%)
Absent	263 (46.1%)
*Post-delivery age preceding the survey *	
< 6 month	235(41.2%)
6-12 months	335(58.8%)
*Antenatal care visit at least once*	*164 (28*%)
*Place of birth *	
Home	470 (82.5%)
Health center	64 (11.2%)
Hospital	10 (1.8%)
Health post	26 (4.6%)
*History of obstetric complication*	165 (28.9%)
*Types of obstetric complications (n=401)*	
Vaginal bleeding	74 (18.0%)
A severe headache	88 (21.5%)
Blurred vision	86 (21.0%)
Swollen hands/face	35 (8.5%)
Loss of consciousness	20(4.9%)
Prolonged labor (>12 hours)	66 (16.1%)
Retained placenta	33(8.0%)
Other complications	8(2.0%)
Total complications	410 (100%)

**Table 3:** Knowledge of obstetric danger sign among study participants in Jardega Jarte District, Oromia Region, Western Ethiopia, from January 1 to February 2, 2016.

Response and exact identification of danger signs	Frequency (%)
Reported as they know danger sign during pregnancy (N=570)	397 (69.5%)
Abel to identify the three key pregnancy danger signs (n=397)	125 (31.48%)
Reported as they know danger sign during labor and birth (N=570)	344 (60.4%)
Abel to identify the four key danger signs during labor and birth (n=344)	67 (19.5%)
Reported as they know danger sign during post-partum period (N=570)	313 (54.9%)
Abel to identify the three key post-partum danger signs (n=313)	179 (57.2%)

**Table 4:** Level of birth preparedness from a study conducted among women who gave birth recently in Jardega Jarte district Oromia Region, western Ethiopia, from January 1 to February 2, 2016.

Variables		Frequency (%)
Respondents report on birth preparedness	Yes	395 (69.3%)
	No	175 (30.7%)

Birth preparedness*∗*	Well prepared	157(27.5%)
	Less prepared	413(73.5%)

*∗*According to this study, a woman was said to be well prepared for birth preparedness if she has *identified health facility for emergency and normal delivery, arranged transportation, and saved money for her recent delivery*.

**Table 5:** Predictor variables of birth preparedness in multivariable analyses among study participants in Jardega Jarte District, Oromia Region, western Ethiopia, from January 1 to February 2, 2016.

Characteristics	Well prepared	Less prepared	COR	AOR
	No (%)	No (%)	(95 % CI)	(95 % CI)
*Residence *				
Urban	31(54.4)	26(45.6)	3.66 (2.09-6.4)	3.44 (1.7-6.9)*∗*
Rural	126(24.6)	387(57.4)	1.00	1.00
*Presence of under-five child*				
Yes	41(13.4)	266(86.6)	0.195 (0.13-0.29)	0.207 (0.12-0.35)*∗*
No	116(44.1)	147(55.9)	1.00	1.00
*Past obstetric complications*				
Yes	88(53.3)	77 (46.7)	5.56 (3.7-8.3)	4.05 (2.26-7.75)*∗*
No	69(17)	336 (83)	1.00	1.00
*Antenatal care visit*				
Yes	88(53.3)	76 (46.3)	5.65 (3.78-8.4)	2.9 (1.67-5.16)*∗*
No	69(17)	337 (83)	1.00	1.00
*Parity*				
Primipara	41(64.1)	23 (35.9)	9.85 (5.34-18.1)	5.12 (2.4-10.8)*∗*
Multipara (2-4)	76(31)	169 (69)	2.48 (1.6-3.83)	2.3 (0.99-3.85)
Grand multipara (≥5)	40(15)	221(84)	1.00	1.00
*Fixed income source *				
Yes	74 (51.4)	70(48.6)	4.37 (2.9-6.5)	0.33 (0.03, 4.17)
No	83 (19.5)	343(80.5)	1.00	1.00
*Husband occupation*				
Other than farmer	55(35.9)	53(13.6)	3.57 (2.3-5.37)	0.21 (0.12-1.54)
Farmer	98 (64.1)	337(86.4)	1.00	1.00
*Husband age*				
20-29	72(45.0)	88(55)	6.17 (3.06-12.45)	0.41 (0.04, 4.38)
30-39	70(24.2)	219(75.8)	2.41 (1.22-4.78)	0.28 (0.02, 3.23)
≥40	11(11.7)	83(88.3)	1.00	1.00

*∗*Variables showing statistical significance at P value of <0.05.

## Data Availability

The datasets analyzed during the current study are available from the corresponding author on reasonable request.
